# Real-time monitoring of drug pharmacokinetics within tumor tissue in live animals

**DOI:** 10.1126/sciadv.abk2901

**Published:** 2022-01-07

**Authors:** Ji-Won Seo, Kaiyu Fu, Santiago Correa, Michael Eisenstein, Eric A. Appel, Hyongsok T. Soh

**Affiliations:** 1 Department of Electrical Engineering, Stanford University, Stanford, CA 94305, USA.; 2 Department of Radiology, School of Medicine, Stanford University, Stanford, CA 94305, USA.; 3 Department of Materials Science and Engineering, Stanford University, Stanford, CA 94305, USA.

## Abstract

The efficacy and safety of a chemotherapy regimen fundamentally depends on its pharmacokinetics. This is currently measured based on blood samples, but the abnormal vasculature and physiological heterogeneity of the tumor microenvironment can produce radically different drug pharmacokinetics relative to the systemic circulation. We have developed an implantable microelectrode array sensor that can collect such tissue-based pharmacokinetic data by simultaneously measuring intratumoral pharmacokinetics from multiple sites. We use gold nanoporous microelectrodes that maintain robust sensor performance even after repeated tissue implantation and extended exposure to the tumor microenvironment. We demonstrate continuous in vivo monitoring of concentrations of the chemotherapy drug doxorubicin at multiple tumor sites in a rodent model and demonstrate clear differences in pharmacokinetics relative to the circulation that could meaningfully affect drug efficacy and safety. This platform could prove valuable for preclinical in vivo characterization of cancer therapeutics and may offer a foundation for future clinical applications.

## Teaser

A biosensor that can continuously monitor drug levels in vivo at multiple tumor sites in a live rodent model is demonstrated.

## INTRODUCTION

Interindividual differences in pharmacokinetics (PK) can profoundly affect the efficacy of drug treatment, particularly in the context of chemotherapy for cancer (*1*, *2*). This variability makes it challenging to identify the appropriate therapeutic window for a given drug regimen. Underdosing reduces the likelihood of successful treatment, whereas overdosing can inflict severe damage on the kidney, liver, heart, and other organs (*3*–*7*). Currently, such PK data are typically collected via blood-based measurements of circulating drug concentrations, and a number of groups have even demonstrated the feasibility of real-time drug monitoring through the use of miniaturized implantable sensors (*8*–*11*). However, such circulation-based measurements do not adequately reflect drug absorption within the tumor tissue itself (*12*–*16*). This is because the microenvironment within tumors is complex, with unpredictable vascular permeability, heterogeneous and high interstitial fluid pressure, high cell density, and disorganized lymphatic drainage (*17*–*19*). These factors can collectively impede the continuous and homogeneous penetration of drugs from plasma into the tumor tissue, resulting in notable differences in drug concentration between the plasma and different regions of the tumor. Hence, measurements of drug concentrations in plasma can yield inaccurate assessments of PK, resulting in low therapeutic efficacy.

Currently, the only way to obtain tumor-specific PK measurements is through tissue specimens collected via needle-based biopsies. However, it is difficult to extrapolate the overall drug penetration in the heterogeneous tumor tissue environment from a single sampling site, and this, in turn, leads to inaccurate PK assessment. Multiple biopsies would offer a more complete picture of tumor PK, but it is impractical to perform this invasive procedure repeatedly, because it is costly, time consuming, and carries the risk of severe side effects—including tumor seeding (*20*–*23*). Even in animal models, multiple biopsies of tumors are technically challenging, and so researchers typically carry out PK studies by collecting samples from multiple animals sacrificed at different time points, producing averaged population data that do not accurately capture intratumor variability from individual animals (*24*). Furthermore, these experiments are being performed ex vivo and may not accurately capture the physiological behavior of a tumor within its in vivo milieu. More generally, such analyses are challenging to perform for a variety of reasons—for instance, the probe needs to withstand insertion into solid tissue with minimal sensor damage and must be sufficiently resistant to biofouling to enable robust measurement over extended periods of time. Accordingly, there remains an unmet need for analytical tools that are capable of efficiently collecting accurate PK data from multiple tumor sites simultaneously.

Here, we demonstrate an electrochemical aptamer-based biosensor that enables robust, real-time, multisite drug monitoring within tumor tissue in live animals. Our biosensor features a number of technical and design advances that enable it to overcome key limitations that have hindered past efforts to achieve drug monitoring within solid tissues (*25*–*29*). First, we make use of nanoporous gold microelectrodes that successfully minimize both the effects of fouling from biological matrices and the risk of sensor damage from tissue insertion. Second, each sensor incorporates several such microelectrodes so that we can monitor drug concentrations with a higher signal-to-noise ratio (SNR) at multiple sites within tumor tissue simultaneously. Last, our sensor is flexible, offering a better match to the physical properties of surrounding tissue and thereby minimizing damage at the site of insertion. As a demonstration, we show that our microelectrode array sensor can monitor concentrations of the chemotherapy drug doxorubicin (DOX) at multiple positions in tumor tissue simultaneously. This enables us to collect in situ tumor–specific PK data that account for tissue heterogeneity within a single animal, revealing patterns of DOX distribution within the tumor tissue that differ starkly from those measured via the systemic circulation. These differences indicate that the latter metrics might prove misleading in the selection of an appropriate drug dose and highlight the importance of in situ PK monitoring in the context of cancer therapeutics research. Our data indicate that our biosensor platform could offer a simple and robust tool for obtaining more physiologically relevant insights into drug PK and understanding the in vivo behavior of experimental drugs.

## RESULTS AND DISCUSSION

### Overview and fabrication of the sensor

**Fig. 1.**
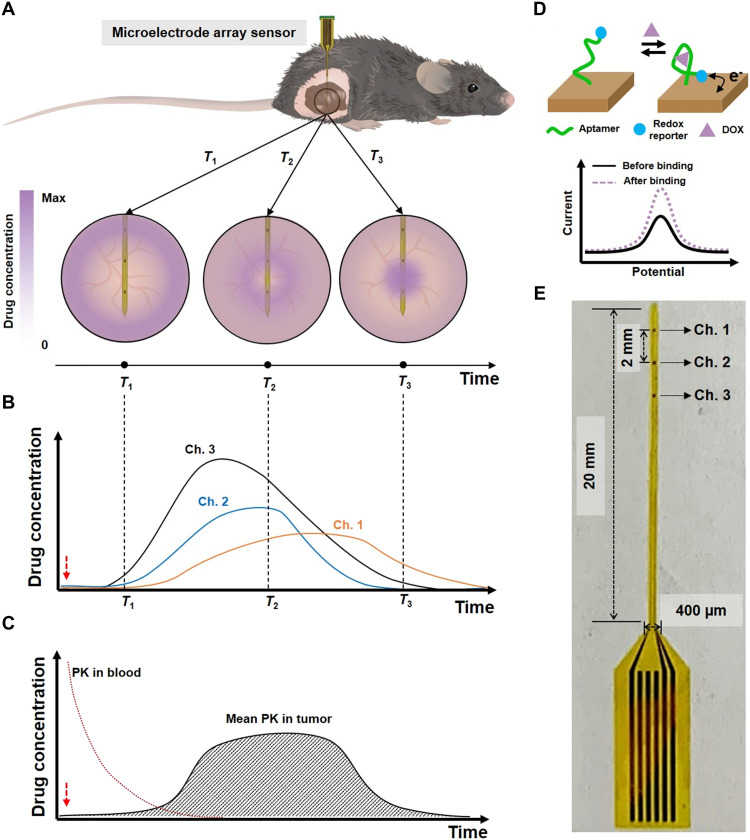
Gold nanoporous microelectrode array–based implantable electrochemical aptamer sensor. (**A**) Illustration of sensor implantation into tumor tissue in a live mouse (top) and detecting drug concentrations within the tumor at different time points (*T*_1_, *T*_2_, and *T*_3_; bottom). (**B**) Illustration of real-time measurement of different drug concentrations at each microelectrode channel. Dashed lines represent the time points from (A). (**C**) Illustration of averaged real-time drug concentrations from the three sensor channels versus drug concentrations from the blood. Red dashed arrows in (B) and (C) indicate the time of drug injection. (**D**) Schematic of the aptamer-based drug detection mechanism. (**E**) Photo of our sensor.

Our sensor comprises an array of gold nanoporous microelectrodes integrated into a flexible, polyimide (PI) polymer–based probe, which can be implanted directly into tumor tissue in a live mouse (Fig. 1A). This sensor is designed to collect temporal drug concentration profiles at multiple sites within tumor tissue simultaneously via multiple microelectrodes in real time (Fig. 1B). On the basis of the resulting measurements, the PK of the drug can then be assessed in terms of its absorption and elimination phases, where the former refers to the drug’s uptake into the tissue from the circulatory system and the latter describes the drug’s subsequent clearance from the tissue due to lymphatic drainage and other physiological processes (*30*, *31*). The mean intratumoral PK of the drug—as calculated based on concentration data obtained at three different microelectrode channels—can then be compared to the systemic PK (Fig. 1C).

Detection is achieved by functionalizing the surface of these gold microelectrodes with aptamers for the drug of interest that undergo a conformational change upon binding to their target. For the present work, we used a well-characterized aptamer that can bind DOX—a widely used chemotherapeutic drug (*32*–*36*). As described in our previous work (*37*), the distal end of the aptamer is tagged with a methylene blue (MB) redox reporter; in the presence of the target, the aptamer undergoes a conformational change that increases the rate of electron transfer between the MB reporter and the electrode surface, thereby yielding an increase in current (Fig. 1D). We applied square wave voltammetry (SWV) to determine the signal gain, which is the ratio of current measurements from before and after target addition. Since the aptamer can reversibly bind and release its target, our sensor can continuously measure target concentration and kinetic information in real time.

We recently reported that nanoporous structured electrodes offer greatly improved electrochemical detection sensitivity relative to planar electrodes, with a higher SNR due to reduced charge screening effects (*38*). This enhanced sensitivity is important in this context, because in vivo experiments intrinsically have high background noise. The use of nanoporous microelectrodes should also facilitate long-term monitoring in tumor tissue, because aptamers residing within the nanopores are better protected against both mechanical damage during insertion and biofouling during implantation (*39*, *40*). Each sensor comprises an array of several such nanoporous gold microelectrodes fabricated onto a flexible PI substrate, which forms a 400 μm × 20 mm shank with a thickness of 15 μm (Fig. 1E and fig. S1). The entire fabrication process is detailed in the Supplementary Materials (fig. S2). Briefly, an Au-Ag alloy film was deposited onto the PI substrate by cosputtering of Au and Ag, after which the silver was dissolved in 69% nitric acid (fig. S3A). To prevent degradation of the PI substrate during this process, we added a gold bottom protective layer before deposition of the Au-Ag alloy (fig. S3B). We then functionalized these microelectrodes with the MB-tagged DOX aptamer. For this work, we used a three-channel array consisting of 100 μm × 100 μm microelectrodes positioned with a 2-mm pitch. This pitch enables the sensor to measure a large area of tumor tissue simultaneously, while the relatively small area of the microelectrodes confers high spatial resolution compared to needle biopsies or metal wire–based sensors. The sensor was connected to a printed circuit board (PCB), which was, in turn, connected to a commercial potentiostat (fig. S4). The working electrode array in the sensor and a conventional Ag/AgCl reference electrode were used together for all measurements. After recording the SWV curves with the potentiostat, we used a custom MATLAB script to calculate the DOX concentration.

The mechanical flexibility conferred by our PI substrate is another essential feature of our in vivo sensor. It is well known that sensors made of silicon or metal wires lead to a mismatch in mechanical properties between the sensor (stiffness ~1 mN·m) and surrounding tissue (~100 nN·m) (*41*), inflicting damage on the tumor tissue. Such tissue damage could lead to inaccurate measurement, prevent long-term monitoring, or cause additional tumor seeding (*22*). Our flexible probe minimizes such risks, because its stiffness is sufficiently low (~270 nN·m) to approach that of tumor tissue (*42*–*44*).

### Characterizing sensor performance of our microelectrode array

**Fig. 2.**
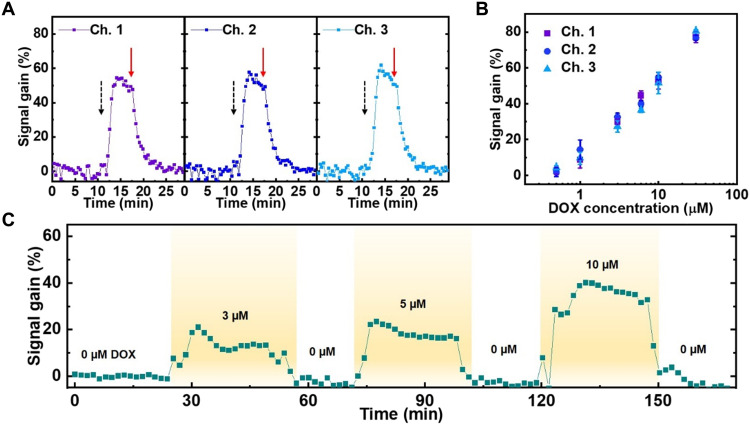
Assessing the consistency and reproducibility of microelectrode array measurements. (**A**) Continuous measurements of signal gain from each of our three channels at baseline after adding a 10 μM bolus of DOX at *t* = 12 min (black dashed arrows) and after washing in 1× SSC buffer at *t* = 17 min (red solid arrows). (**B**) Signal gain from all three microelectrode channels at increasing DOX concentrations. Error bars were calculated from five different data points at each concentration. (**C**) Continuous measurements of signal gain in flowing fetal bovine serum (FBS) containing different concentrations of DOX. SWV frequency = 200 Hz and amplitude = 50 mV. The data represent average signal gain from the continuously measured signal gain data collected at the three channels.

We initially tested the performance of our sensors in a series of in vitro experiments. We first confirmed that each microelectrode in our array has equivalent sensitivity. Briefly, we immersed our sensor in 1× saline sodium citrate (SSC) buffer and introduced 10 μM DOX after allowing the baseline signal to stabilize for 12 min. All three channels showed a similar response, producing an average ~53.9% signal gain with 1.4% variance between channels (Fig. 2A). At *t* = 17 min, we washed the sensor, and the signal returned to baseline within minutes, confirming the capacity for continuous, real-time sensing. We next exposed our multichannel sensor to increasing DOX concentrations and observed a clear and proportional signal gain; our three electrodes exhibited a steady increase in signal gain from 2.9% at 500 nM to 78.1% at 30 μM, with just 3% variance in signal gain at each concentration across the three electrodes (Fig. 2B). We calculated the average signal gain of each DOX concentration from the data obtained at the three electrodes and then performed curve fitting for this average signal gain data after plotting versus DOX concentration (fig. S5). We used this curve for the calibration of DOX concentration from the signal gain in all ex vivo and in vivo measurements described throughout the manuscript.

We next set out to characterize the performance of our sensor in fetal bovine serum (FBS). We positioned our microelectrode array sensor vertically within a polydimethylsiloxane (PDMS) chamber, through which we continuously flowed undiluted serum with a peristaltic pump (fig. S6). We increased the DOX concentration in serum to 3, 5, or 10 μM at different time points and maintained each condition for 30 min. The aptamer-functionalized microelectrodes clearly responded to each DOX concentration, producing signal gains ranging from 13.2% at 3 μM DOX to 38.8% at 10 μM DOX (Fig. 2C). The sensor consistently returned to baseline when DOX was no longer present in serum, even after nearly 3 hours of continuous data collection—a time scale that is standard for clinical DOX administration.

**Fig. 3.**
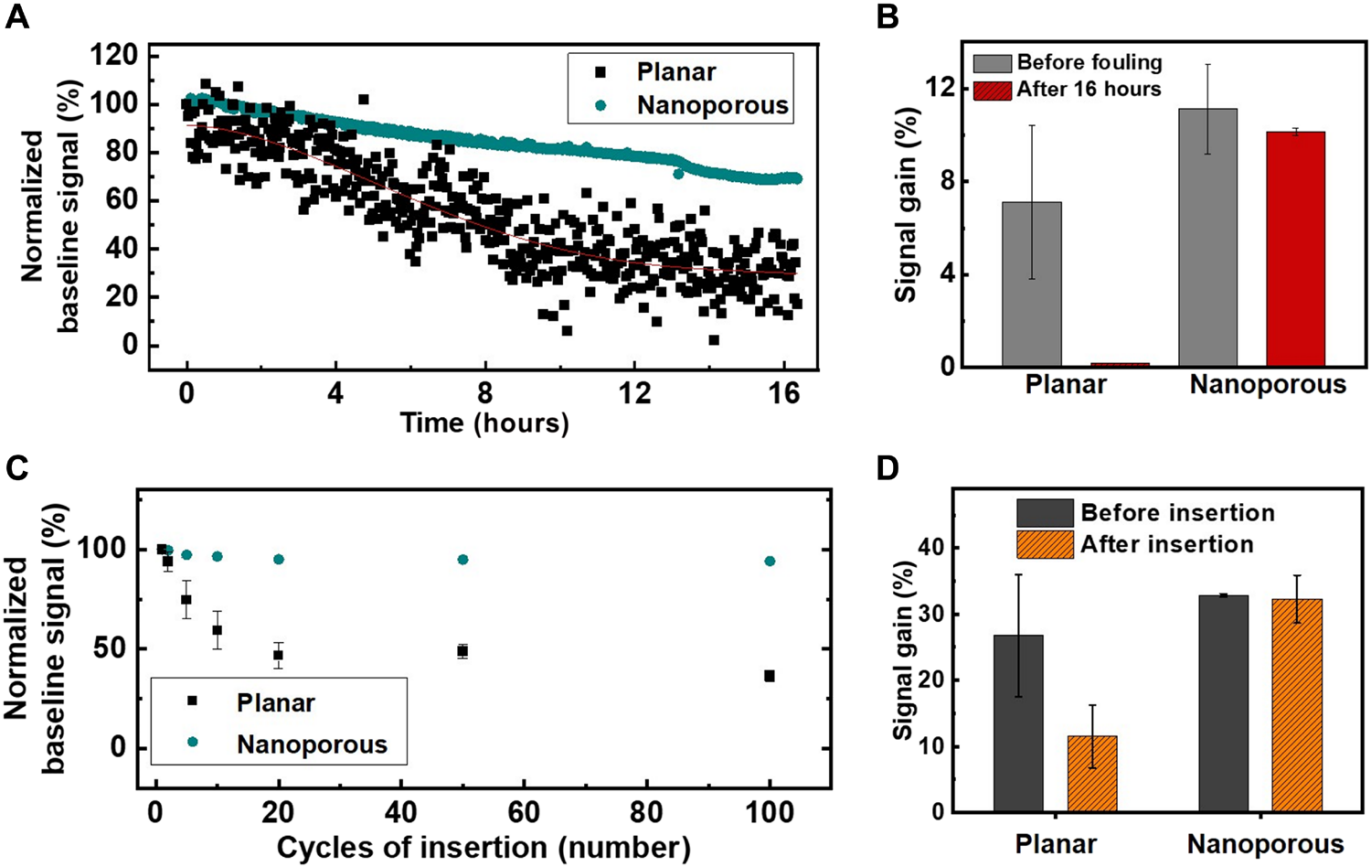
Stability of sensor performance in serum and tissue. (**A**) Representative data from a single channel showing baseline signal on gold nanoporous and planar microelectrodes over the course of 16 hours in undiluted FBS. (**B**) Average signal gain (*n* = 2 channels) produced by gold nanoporous and planar microelectrodes in the presence of 3 μM DOX in serum upon initial exposure (gray) and after 16 hours in serum (red). (**C**) Averaged baseline signal change (*n* = 3 channels) for gold nanoporous and planar microelectrodes after multiple cycles of insertion into murine melanoma tumor tissue. (**D**) Averaged signal gain (*n* = 3 channels) from 6 μM DOX in 1× SSC on planar and nanoporous microelectrodes before initial insertion into tumor tissue and after 100 insertion cycles. SWV frequency = 200 Hz and amplitude = 50 mV.

Biofouling of electrode surfaces poses a major problem for in vivo detection, and the adsorption of proteins present in the blood around and inside tumor tissue can render sensors unusable after a short period. Previous aptamer-based sensors have used a passivation layer on the electrode surface to mitigate this problem, but even with such measures, most aptamer-based sensors to date have reported a functional lifetime of no more than 12 hours in flowing blood (*45*–*47*). On the basis of prior work with nanostructured gold microelectrodes (*39*, *40*), we anticipated that our sensor would enable stable, long-term monitoring due to sequestration of the aptamers within nanopores, minimizing the effects of biofouling. To verify this, we measured the baseline signal of both planar and nanoporous microelectrodes in flowing serum for 16 hours. As expected, the signal from planar microelectrodes worsened over time and decreased to 10% of the baseline signal by 16 hours, indicating severe biofouling and/or degradation of the aptamer (Fig. 3A). In contrast, the nanoporous microelectrodes maintained 70% of the baseline signal after 16 hours, indicating much more stable sensor performance in conditions that are highly conducive to biofouling. We next assessed the signal gain produced in response to a 3 μM DOX spike in serum—a standard clinical dose—at time zero versus after 16 hours exposure to serum. At initial exposure, the nanoporous microelectrode produced an average signal gain of 11.1 versus 7.1% for the planar microelectrodes (Fig. 3B and fig. S7). After 16 hours, the signal gain from the nanoporous electrodes decreased only slightly to 10.1% at 3 μM DOX, whereas the planar electrode sensor no longer produced a measurable signal gain. These results strongly suggest that the nanoporous gold microelectrodes are far less susceptible to biofouling and/or degradation of the DOX aptamer and therefore better suited for long-term in vivo measurements.

### Measurements from gold nanoporous microelectrodes inserted into tissue

We also anticipated that our nanoporous structured electrodes would offer protection against mechanical damage to the sensor surface, which could otherwise result in unstable and reduced signal after insertion. For comparative purposes, we fabricated three-channel planar microelectrode array sensors that were otherwise identical to our nanoporous microelectrode array sensors. We inserted planar and nanoporous gold microelectrode array sensors into tumor tissue and analyzed changes in the baseline signal over the course of multiple cycles of insertion. For these experiments, we used tumor tissue derived from the murine B16-F10 melanoma model, which was grown subcutaneously in the hind flank of a C57BL/6 mouse (*48*). In each insertion cycle, the sensor was inserted into tumor tissue and withdrawn immediately. The signal was then measured in 1× SSC buffer after each cycle of insertion. After 10 cycles, the average signal from planar microelectrodes decreased to 59.4% of the original baseline signal, and after 100 cycles, this signal was reduced to 36.4% of baseline (Fig. 3C). In contrast, our nanoporous microelectrodes retained 94.1% of their baseline signal even after 100 cycles of insertion. These results clearly demonstrate that the gold nanoporous microelectrodes are very well suited for electrochemical detection in the context of solid tissue, with minimal mechanical damage after insertion to either the substrate or the functionalized aptamers.

We further confirmed this result by comparing the signal gain in response to 6 μM DOX in 1× SSC for these various microelectrodes before insertion into tumor tissue and after 100 cycles of insertion (Fig. 3D). The planar and nanoporous microelectrodes respectively showed comparable average signal gain of 26.8 and 32.8% before insertion. But after 100 cycles, the signal gain from the planar microelectrodes sharply decreased to 11.5%, whereas the nanoporous electrode still maintained a 32.3% signal gain. We also assessed how well our probe performed in the context of tissues with different mechanical properties and found that our nanoporous microelectrode sensor retains its baseline signal independent of the elastic modulus of the tissue environment (fig. S8), confirming the broad mechanical compatibility of our sensor design.

### Ex vivo real-time monitoring of DOX

**Fig. 4.**
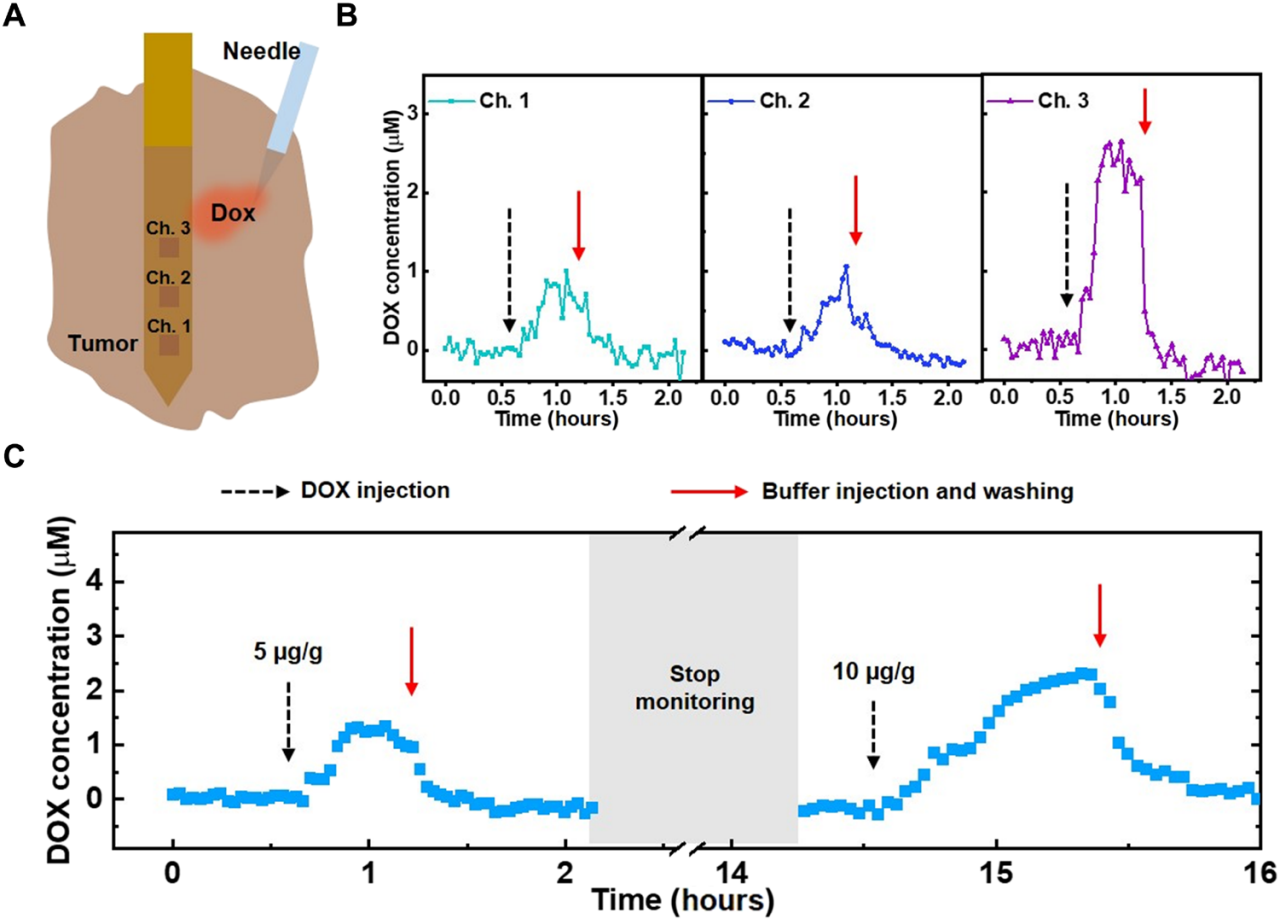
Ex vivo monitoring of DOX in tumor tissue. (**A**) The sensor was implanted into extracted tumor tissue, after which a bolus of DOX was injected. (**B**) Real-time DOX concentration at each channel after injecting a bolus of DOX (5 μg/g) (black dashed arrow) at a tumor site near channel 3, followed by washing with 1× SSC buffer (red solid arrow). (**C**) Averaged real-time DOX concentrations from the three sensor channels in tumor tissue over the course of 16 hours after spiking in boluses of DOX (5 or 10 μg/g) at different time points. SWV frequency = 200 Hz, amplitude = 50 mV, *n* = 3 channels.

We next assessed the real-time, multichannel DOX detection capabilities of our sensor in surgically removed tumor tissue (Fig. 4A). We extracted **~**100 mm^2^ of B16-F10 tumor tissue grown in a C57BL/6 mouse. After transferring the tumor tissue to a PDMS chamber within a small volume of buffer, we implanted our sensor into the middle of the tumor tissue such that all three channels of the array were embedded, with channel 1 closest to the center of the tissue. We then injected a bolus of DOX (5 μg/g) adjacent to channel 3 and measured the DOX concentration at the three microelectrode channels (Fig. 4B). All three clearly responded to this DOX spike in real time, but each channel detected a different concentration. Channel 3, which was closest to the injection site, detected an average ~2.4 μM DOX, whereas the more distal channels 1 and 2 detected much lower average concentrations of ~0.8 and 0.7 μM, respectively. We next injected 500 μl of 1× SSC buffer three times into the tumor to wash the drug out completely, and this treatment returned the measured concentration to zero. These results demonstrate that our system can discriminate spatial differences in the drug concentration profile at different positions within the tumor tissue.

We were also able to achieve continuous detection for extended periods of time within tumor tissue. We injected boluses of DOX at a tumor site adjacent to the sensor at different time points and monitored the DOX concentration over the course of 16 hours (Fig. 4C). After applying the first bolus (5 μg/g), we recorded a peak DOX concentration of 1.3 μM, which returned to zero after washing the tumor tissue with SSC. After signal stabilization, we stopped monitoring for 12 hours but left the device implanted; when we restarted monitoring, the signal remained at baseline. We subsequently observed a peak DOX concentration of 2.3 μM after injecting a second bolus of DOX (10 μg/g) at the same site in the tumor. These results demonstrate that our sensor system can achieve robust and stable drug detection even after extended implantation.

### In vivo real-time monitoring of DOX within tumor tissue

Last, we demonstrated the capability to continuously measure drug PK within tumor tissue in a live mouse. We anesthetized a C57BL/6 mouse, which had a 12-mm-diameter B16-F10 tumor, and implanted our sensor such that all three channels were within the tumor tissue, with channel 3 closest to the surface and channel 1 deepest within the tumor tissue (fig. S9). After measuring baseline signal within the tumor tissue, we injected a bolus of DOX (10 μg/g) adjacent to channel 1 and observed the response at all three channels to assess its in vivo real-time recording capabilities (fig. S10). Channels 1 and 2 responded quickly to this DOX injection, while channel 3 did not respond, as it was too far from the injection site for measurable quantities of the drug to diffuse.

**Fig. 5.**
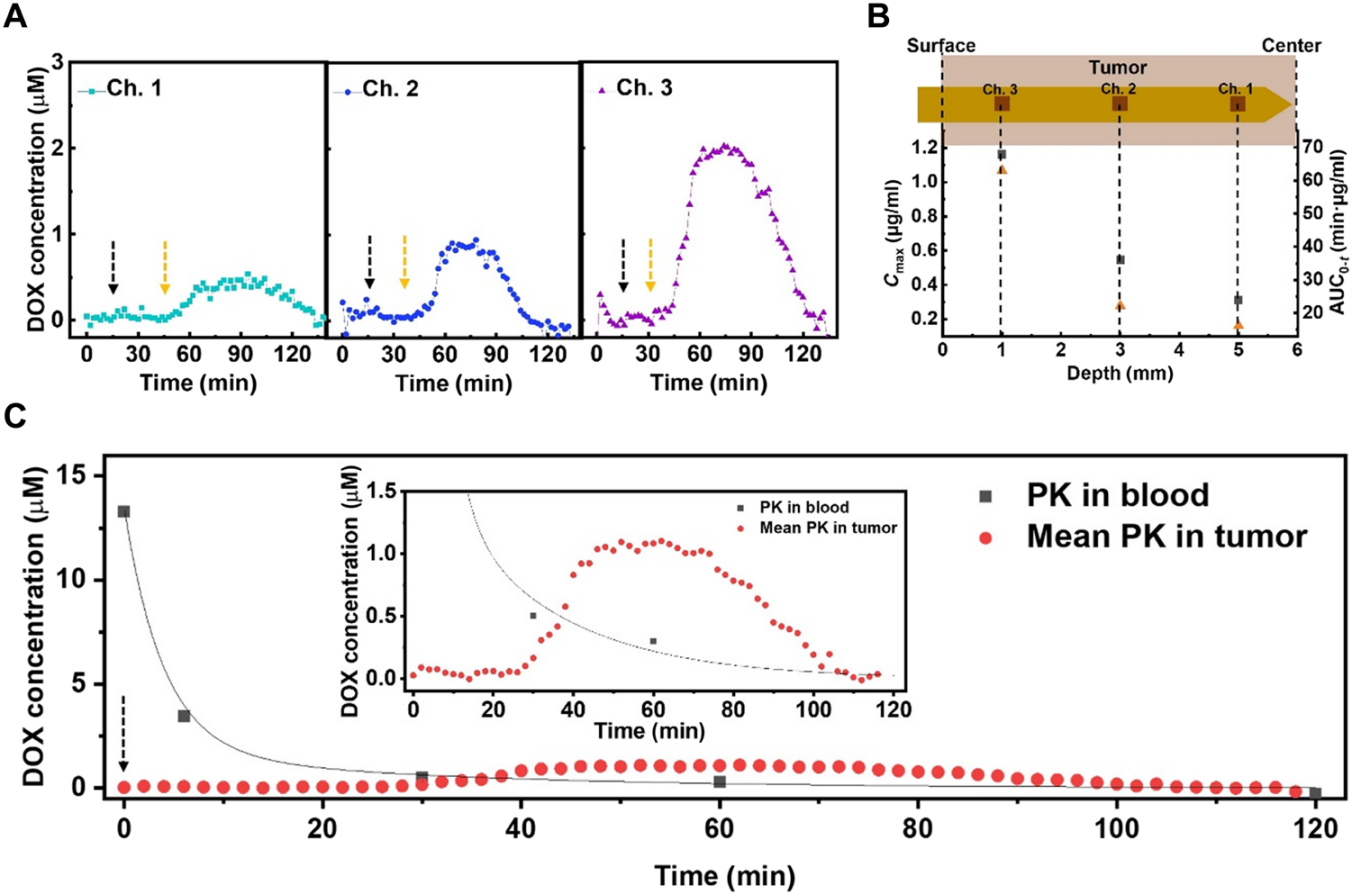
In vivo real-time monitoring of DOX PK in tumor tissue. (**A**) Real-time DOX concentration at each sensor channel after intravenous injection of DOX (10 μg/g) at *t* = 15 min (black dashed arrows). Yellow dashed arrows represent the time points at which the response is observed. (**B**) Maximum drug concentration (*C*_max_) (black square) and area under the curve (AUC_0-*t*_) (orange triangle) as a function of depth within the tumor. (**C**) Averaged real-time DOX concentrations from the three sensor channels versus DOX concentrations obtained from the blood after injecting DOX (10 μg/g) at *t* = 0 min (black dashed arrow). Line represents curve fitting to a two-compartment pharmacokinetic model. Inset shows magnification of averaged real-time intratumoral DOX concentration data. For (A) and (C), SWV frequency = 200 Hz and amplitude = 50 mV.

We next anesthetized a second C57BL/6 mouse, which also had a 12-mm-diameter B16-F10 tumor, and implanted another sensor in the same manner described above. We collected the blood from the tail vein using a heparinized capillary tube and inserted an additional third sensor inside the tube. After measuring the baseline signal within the tumor tissue and circulation, we intravenously injected DOX (10 μg/g) through the tail vein. We observed different DOX concentration profiles from each of the three channels in the intratumoral probe (Fig. 5A), indicating that the drug PK exhibits considerable site-dependent variability due to factors including irregular microvasculature density and interstitial fluid pressure across the whole of the tumor tissue (*15*, *49*–*51*). In parallel, we collected blood samples from the tail vein at 0-, 5-, 30-, 60-, and 120-min time points after the intravenous injection to measure circulatory PK.

Channel 1 was nearest the center of the tumor, where the high interstitial fluid pressure due to the dense microvasculature results in a lower rate of drug penetration relative to the surface of tumor tissue (*52*, *53*). We derived the PK parameters by fitting to a two-compartment model, which describes the changes in drug concentrations in the central (i.e., systemic circulation) and peripheral (i.e., tumor tissue) compartments (Table 1) (*30*, *54*, *55*). From the perspective of the circulation, this model describes the rate of transport of the drug into the tumor (*K*_d_blood_) and the subsequent rate of drug elimination (*K*_el_) from the body. For the tumor tissue, we derived the PK parameters by fitting to a one-compartment model, which describes the rate of entry of the drug into a particular region of the tumor from the blood (*K*_a_tumor_) and the rate of drug elimination (*K*_el_) from the tumor. Accordingly, we began to observe DOX signal in channel 1 30 min after intravenous injection, followed by a relatively slow increase in drug concentration (*K*_a_tumor_ = 0.05 min^−1^). We observed a much earlier response in channels 2 and 3—18 and 15 min after intravenous injection, respectively—and much higher *K*_a_tumor_ (0.077 and 0.095 min^−1^, respectively).

**Table 1.** Pharmacokinetic parameters in tumor and blood.

PK parameter*	**In blood**	**In tumor**			
		**Channel 1**	**Channel 2**	**Channel 3**	**Mean PK**
AUC_0-*t*_ (min∙μg/ml)	80.915	16.159	22.147	63.057	33.376
*C*_max_ (μg/ml)	7.702	0.312	0.546	1.163	0.025
*T*_max_ (min)	0	79	63	59	64
*t*_1/2_ (min)	3.4	101	82	90	89
*K*_el_ (1/min)	0.138 ± 0.08	0.041 ± 0.005	0.067 ± 0.007	0.04 ± 0.004	0.039 ± 0.004
*K*_a_tumor_ (1/min)	N/A	0.05 ± 0.007	0.007 ± 0.009	0.095 ± 0.009	0.076 ± 0.009
*K*_d_blood_ (1/min)	0.064 ± 0.094	N/A^†^	N/A	N/A	N/A

*AUC_0-*t*_ is the area under the curve until the drug concentration reaches zero; *C*_max_ is the maximum drug concentration; *T*_max_ is the time at *C*_max_; *t*_1/2_ is the elimination half-life; *K*_el_ is the elimination rate of drug; *K*_a_tumor_ is the absorption rate of the drug within the tumor; and *K*_d_blood_ is the distribution rate of the drug from the bloodstream to the tissue.

†N/A, not applicable.

We observed a clear gradient of decreasing drug exposure as we looked deeper into the tumor (Fig. 5B). For example, the maximum drug concentration (*C*_max_) for channel 1 (0.312 μg/ml), which is located at a 5-mm depth within the tumor, was four times lower than for channel 3 (1.163 μg/ml), which is positioned at a 1-mm depth. Similarly, the area under the curve (AUC_0-*t*_), which reflects the total drug exposure over time, gradually decreased from 63.057 to 16.159 min·μg/ml as we sampled deeper within the tumor, which would presumably lead to the differential therapeutic efficacy of chemotherapy across the tumor. In parallel, each channel reached *C*_max_ at an increasingly later time point postadministration (*T*_max_) as we looked deeper and deeper into the tumor (fig. S11). We noted that the PK parameters did not always fall along a clear continuum from channel 1 to 2 to 3. For example, we measured a lower elimination half-life (*t*_1/2_) at channel 2 (82 min) than channel 3 (90 min), although the drug concentration at channel 2 reached *C*_max_ later than channel 3 (*T*_max_ = 63 versus 59 min). We posit that this is due to the fact that the DOX elimination rate (*K*_el_) was higher in the tissue surrounding channel 2 (0.067 min^−1^) compared to channel 3 (0.04 min^−1^). These results reveal how the PK can vary across the tumor tissue in a manner that would be difficult or impossible to measure accurately based on conventional biopsy methods.

Last, we showed that the PK measurements obtained from the tumor tissue differ markedly from the systemic PK (Fig. 5C and Table 1). We made this comparison by determining the mean values for the various PK parameters within the tumor tissue by fitting the average real-time DOX concentrations at all three channels over the course of the experiment. We observed that the total drug exposure (AUC_0-*t*_) in the blood (80.915 min·μg/ml) was ~2.5-fold greater than in the tumor (33.376 min·μg/ml), indicating that the tumor received only a fraction of the drug circulating in the blood. Likewise, *C*_max_ in the blood (7.702 μg/ml) was more than 10-fold greater than in tumor tissue (0.625 μg/ml), which raises the possibility of suboptimal drug dosing if one was to rely entirely on PK data from the bloodstream. The *t*_1/2_ and *K*_el_ values were respectively very short (3.4 min) and fast (0.138 min^−1^) in the blood, reflecting rapid clearance by renal excretion (*56*). In contrast, elimination was considerably slower within the tumor (*t*_1/2_ = 89 min, *K*_el_ = 0.039 min^−1^). This indicates that the drug remains present and active within this tissue for a longer duration, which could result in an overdose if one were to rely solely on PK measurements from the blood. Conversely, this slower elimination rate could result in a more potent effect even from a seemingly low dose, which is a useful consideration when attempting to identify the appropriate therapeutic window. This notable variability in PK parameters between the blood and tumor demonstrates the importance of obtaining local measurements of drug absorption within tumor tissue and shows how in situ tumor measurements collected with our sensor could facilitate the selection of more effective and appropriate drug dosing strategies.

Drug PK can vary considerably within cancerous tissue relative to the systemic circulation due to the abnormal physiology of the tumor microenvironment. As a consequence, blood-based measurements of drug absorption and elimination are likely to produce misleading or inaccurate PK measurements that impede efforts to identify an optimally safe and effective dose for cancer therapeutics. In this work, we present a microelectrode array–based implantable electrochemical aptamer sensor that overcomes this problem by enabling simultaneous multisite drug concentration monitoring within tumor tissue in real time. Our sensor incorporates gold nanoporous electrodes that are highly resistant to both damage and biofouling during or after tissue implantation and is built on a flexible polymer substrate that matches the physical properties of the surrounding tissue and thus minimizes tissue disruption at the site of insertion. Each probe contains three distinct microelectrode channels, and we demonstrate the capacity to sensitively discriminate local differences in the intratumoral concentration of DOX at each channel. Our sensor enabled us to collect extensive tumor-specific PK data over the course of multiple hours that reveal notable differences in the profile of DOX absorption and elimination relative to PK measurements based on systemic circulation from the same animal. Our intratumoral PK measurements are comparable to previous literature that reported intratumoral measurements obtained by extracting the tumors from animals at different time points (table S1) (*57*–*59*).

On the basis of these findings, we believe that our sensor platform could offer a highly effective tool for preclinical analysis of the PK characteristics of experimental drugs, thereby guiding dose selection for first-in-human studies that maximize likelihood of efficacy while minimizing dose-related toxicity. The foundational sensor design demonstrated here should be readily extensible to include larger numbers of channels that can produce measurements with even greater spatial resolution. The current sensor is still limited in terms of temporal resolution and could not achieve detection in the time scales required for probing extremely rapid phenomena such as monitoring neurotransmitter release in the brain. However, this system should be very well suited for applications such as drug and biomarker monitoring in the blood in preclinical studies. In principle, different microelectrode channels could also be functionalized with different aptamers, enabling the real-time monitoring of multiple drug agents in the context of a combination therapy, or simultaneous measurement of a therapeutic agent and a biomarker related to drug response. With further refinement and demonstration of the long-term stability, safety, and biocompatibility of this probe design, we could envision future adaptations of this platform for potential use in clinical drug and biomarker studies.

## METHODS

### Reagents and materials

The DOX aptamer was obtained from Biosearch Technologies: 5′-HS-C6-ACCATCTGTGTAAGGGGTAAGGGGTGGT-MB-3′, where MB indicates the methylene blue redox reporter. Tris(2-carboxyethyl)phosphine (TCEP), DOX, and 6-mercapto-1-hexanol (6-MCH) were purchased from Sigma-Aldrich. A diluted 1× SSC buffer was prepared by diluting 20× SSC buffer (Thermo Fisher Scientific) with nuclease-free water. FBS was obtained from Thermo Fisher Scientific. Solutions of various DOX concentrations were prepared by dissolving DOX in either 1× SSC buffer or undiluted FBS. The conventional Ag/AgCl reference electrode was prepared from 500-μm-diameter Ag wire (41390 Silver wire, Alfa Aesar) treated with 1 M iron(III) chloride (Sigma-Aldrich) solution for 1 min.

### Fabrication of microelectrode array sensor

A schematic of the device fabrication process is shown in fig. S2. For the planar gold microelectrodes, a 300-nm-thick aluminum (Al) sacrificial layer was deposited on a Si wafer by electron beam evaporation (ATC-E, AJA International Inc.). A 15-μm-thick PI layer (PI2574, HD Microsystems) was spin-coated onto the Al layer by manual resist spinner (Headway Research) and thermally imidizated by baking in a N_2_-purged oven at 250°C for 2 hours and subsequently cooling down to room temperature (RT) for 4 hours. Then, a 2-μm-thick negative photoresist (PR) layer (NR9-3000PY, Futurrex) was spin-coated onto the PI layer and then patterned by photolithography with a contact mask aligner (Karl Suss MA6, SÜSS MicroTec) to make the pattern of a 100 μm × 100 μm three-channel array. Subsequently, a Ti/Au (5/70 nm) layer was deposited onto the PR layer using electron beam evaporation, after which the gold planar microelectrode array was formed through a lift-off process in which the PR layer was dissolved in acetone.

For the nanoporous microelectrode arrays, a 4.5-μm-thick positive PR layer (MEGAPOSIT SPR220-3, Kayaku Advanced Materials) was subsequently spin-coated onto the sample. This was patterned to make the pattern of a 100 μm × 100 μm three-channel array as described above. A Ti/Au (10/50 nm) bottom protective layer was then deposited onto the PR via sputter deposition (LAB Line SPUTTER, Kurt J. Lesker Co.). A 300-nm-thick Au-Ag alloy layer was then deposited by cosputtering Au and Ag. The alloy was composed of 66.7% Ag and 33.3% Au. After deposition, the sample was immersed in 69% (v/v) nitric acid for 7 min at RT to dissolve the Ag, forming a gold nanoporous layer. A lift-off process was then used to form the final gold nanoporous microelectrode array.

For both sensor designs, a 2-μm-thick SU8 encapsulation layer (SU8-2002, Microchem) was spin-coated onto the sample and then patterned by photolithography with the contact mask aligner to encapsulate the entire sensor with the exception of the microelectrode array. This assembly was then dry-etched with an ICP-RIE etcher (Versaline LL ICP, Plasma-Therm) and Al etching mask to define the shape of the sensor. After removal of the Al etching mask, the sensor was released from the wafer through anodic dissolution of the Al sacrificial layer. For this step, the Si wafer was connected to a DC power supply (1666, B&K Precision Corporation), immersed in 2 M NaCl, and 15-V DC voltage was applied to the Al layer.

### Functionalization of aptamer onto the electrode

The DOX aptamer was dissolved in nuclease-free water with a concentration of 100 μM. This solution was reacted with a 1000-fold molar excess of TCEP solution with a 1:1 volume ratio for 1 hour, leading to reduction of the MB moiety and thiol-end group on the aptamer. Afterward, the freshly prepared sensor was rinsed with deionized water and then functionalized with 1 μM TCEP-treated DOX aptamer in 1× SSC buffer for 2 hours at RT. The sensor was then washed with excess buffer and incubated with 7 mM 6-MCH solution for 24 hours at RT to passivate the remaining electrode surface. The sensor was stored in 1× SSC at 4°C until it was used for electrochemical measurement.

### Electrochemical signal measurement

All in vitro measurements were performed in a PDMS chamber with the probe connected to a potentiostat (PalmSens4, PalmSens) and multiplexer (MUX8-R2, PalmSens). The PDMS chamber was made by punching a 6-mm-diameter hole on the 5-mm-thick PDMS film and subsequently putting the PDMS film on a glass slide. We used two types of PCB, one small and one large. Each was soldered with an 8-pin FPC connector (FH19C-8S-0.5SH, Hirose Electric Co.). The other side of the large PCB was soldered to another connector (NPD-FF, Omnetics Connector Corporation). The sensor was connected to the small PCB, which was, in turn, connected to the large PCB. A wire connector (NSD-WD, Omnetics Connector Corporation) connected to the large PCB linked the three channels on the sensor to the commercial potentiostat. We placed the sensor and Ag/AgCl reference electrode into the PDMS chamber and adjusted the height to completely submerge all three channels while avoiding direct contact between the connector and the buffer solution. The PDMS chamber was filled with 1× SSC buffer. SWV measurement was carried out over the potential range of −0.55 to −0.1 V with an amplitude of 50 mV, a step size of 1 mV, and pulse frequencies of 200 Hz. Data processing and visualization were performed with custom MATLAB code. All modules were connected as shown in fig. S4.

Continuous in vitro drug monitoring experiments and biofouling tests in undiluted FBS were conducted in a flowing system that switched between FBS only and FBS plus DOX. Flow was achieved by connecting the PDMS chamber with a peristaltic pump, as shown in fig. S6. The inlet and outlet were connected through fluorinated ethylene propylene tubes (with an inside diameter of 1.58 mm; Tygon tubing), which were then mounted onto the peristaltic pump, with a flow rate of 100 μl/min.

### Melanoma model

All animal studies were performed in accordance with Stanford’s Institutional Animal Care and Use Committee guidelines and protocols (APLAC protocol no. 32947). Murine B16F10 melanoma cells were purchased from the American Type Culture Collection, tested for mycoplasma contamination using the MycoAlert Mycoplasma Kit (Lonza), and cultured with 0.2-μm filtered Dulbecco’s modified Eagle’s medium (Thermo Fisher Scientific) supplemented with 10% FBS (Novus Biologicals) and 1% penicillin-streptomycin (Thermo Fisher Scientific). C57BL/6 mice (7 to 8 weeks old; Charles River Laboratories) were subsequently subcutaneously injected with 100 μl of 3 × 10^6^ B16F10 cells/ml in phosphate-buffered saline above the right hind leg. Following tumor inoculation, mice were monitored for the formation of palpable tumors, which occurred within 7 to 10 days. Tumors were regularly monitored via caliper (Mitutoyu) measurements until they reached the appropriate size for either ex vivo or in vivo studies. Mice were euthanized (CO_2_ asphyxiation followed by cervical dislocation) when overall tumor area (*L* × *W*) exceeded 150 mm^2^ or if mice displayed signs of morbidity (e.g., pain, hunching, ulceration, and wasting).

### Ex vivo tumor experiments

Tumors were surgically removed once they grew to ~100 mm^2^ and a mass of 1 g after euthanasia of the mice. Sensor insertion was tested by inserting the sensor into random positions in the tumor, after which the sensor was immediately withdrawn—this constituted one cycle of insertion. We conducted multiple insertion cycles, and SWV measurements were carried out in 1× SCC buffer after each cycle to monitor sensor function.

DOX detection in ex vivo tissues was assessed by placing excised tumors into the PDMS chamber in a small volume of 1× SSC buffer. The sensor was gently implanted into the middle of the tumor tissue to make sure that all three channels were inside the tissue. SWV measurement was carried out over the potential range of −0.55 to −0.1 V with an amplitude of 50 mV, a step size of 1 mV, and pulse frequencies of 200 Hz as we injected boluses of DOX into the tumor. For ex vivo experiments, we injected a 9.26-μl bolus of 1 mM DOX, representing a drug dose (5 μg/g). Once the measurement was complete, we left the probe in place but washed out the drug by injecting 500 μl of 1× SSC buffer three times into the tumor and collected the waste solution from the PDMS chamber. We then added fresh 1× SSC buffer to the chamber. After signal stabilization, we stopped SWV measurement but left the experimental setup in place for 12 hours. SWV measurement was then carried out again with a second bolus of DOX and an additional washing process.

### In vivo experiments

Tumors were allowed to grow to a sufficient size to accommodate the sensor (~140 mm^2^). Mice were then anesthetized using isoflurane (3% induction and 2% maintenance) and maintained on a heating pad at 35°C in a Faraday Cage (VistaShield, Gamry Instruments). To prevent uneccesary pain or discomfort, mice were subcutaneously injected with buprenorphine SR (sustained release) (0.5 mg/kg), and Puralube ophthalmic ointment was applied to the eyes. Analgesics were given 15 min to take effect before beginning the procedure. Our sensor and Ag/AgCl reference electrode were vertically implanted into the middle of the tumor tissue, and SWV measurement was performed over the potential range of −0.55 to −0.1 V with an amplitude of 50 mV, a step size of 1 mV, and pulse frequencies of 200 Hz.. After stabilization of the baseline signal of the sensor, DOX (10 μg/g) was injected through the tail vein manually using a 28G syringe needle. At the end of the experiments, mice were euthanized.

### PK parameter analysis

The DOX concentration–time curve in the blood (Fig. 5C) was fitted to a biexponential equation, indicating the two-compartment model

$$C_{\text{Doxorubicin}}={\mathrm{Ae}}^{-\alpha t}+{\mathrm{Be}}^{-\beta t}$$ (1)

Where *A* and *B* are maximum plasma concentrations corresponding to the drug distribution phase and drug elimination phase, respectively, and 1/α and 1/β are the half-lives for distribution and elimination, respectively. *C*_Doxorubicin_ is DOX concentration as a function of time.

*K*_d_blood_ was calculated by Eq. 2

$$K_{d\_\text{blood}}=((A*\beta )+(B*\alpha ))/(A+B)$$ (2)

Elimination rate (*K*_el_) was calculated by Eq. 3

$$K_{\text{el}}=(\alpha *\beta )/K_{d\_\text{blood}}$$ (3)

The DOX concentration–time curve within the tumor (Fig. 5, A and C) was fitted to an exponential equation, indicating the one-compartment model

$$C_{\text{Doxorubicin}}={\mathrm{Be}}^{-\beta t}$$ (4)

Where β is *K*_el_ of drug within tumor.

*K*_a_tumor_ was derived by fitting the absorption phase of the DOX concentration–time curve to Eq. 5

$$C_{\text{Doxorubicin}}={\mathrm{Ae}}^{\left(Ka\_\text{tumor}*t\right)}$$ (5)

The absorption phase is the time range from drug injection to when the drug concentration reaches *C*_max_. *C*_max_, *T*_max_, and *t*_1/2_ were directly obtained from the experimental raw data of the DOX concentration–time curve. The AUC was calculated using the definite integral of the DOX concentration–time curve with a time range from drug injection to when the drug concentration reached zero.
