# Supplementary material for: Real-time monitoring of drug pharmacokinetics within tumor tissue in live animals
Source: Sci Adv. 2022 Jan 7;8(1):eabk2901. doi: 10.1126/sciadv.abk2901 (PMC8741190; doi:10.1126/sciadv.abk2901)
Supplement: Supplementary file 1 — Figs. S1 to S11 Table S1 [file sciadv.abk2901_sm.pdf]

Science Advances
AAAS

Supplementary Materials for

Real-time monitoring of drug pharmacokinetics within tumor tissue in live animals

Ji-Won Seo, Kaiyu Fu, Santiago Correa, Michael Eisenstein, Eric A. Appel*, Hyongsok T. Soh*

*Corresponding author. Email: eappel@stanford.edu (E.A.A.); tsoh@stanford.edu (H.T.S.)

Published 7 January 2022, *Sci. Adv.* **8**, eabk2901 (2022)
DOI: 10.1126/sciadv.abk2901

This PDF file includes:

Figs. S1 to S11
Table S1

Supporting information

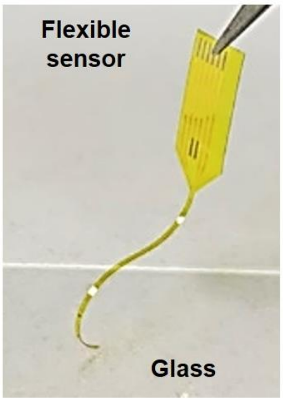

Figure S1. Flexible polyimide-based sensor on glass. The probe is shaped to minimize damage at the site of insertion, and offers a good match to the physical properties of surrounding tissue. Photo Credit: Ji-Won Seo, Stanford University.

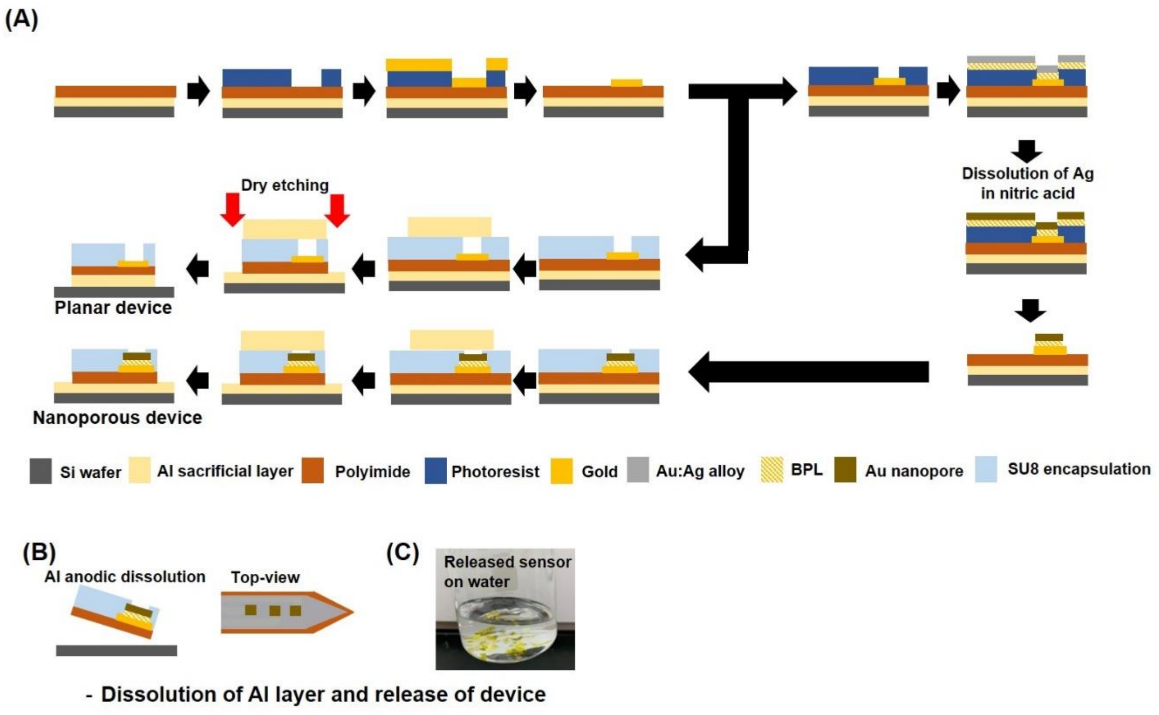

Figure S2. Schematic illustration of the fabrication process of gold nanoporous and planar microelectrode array sensor.

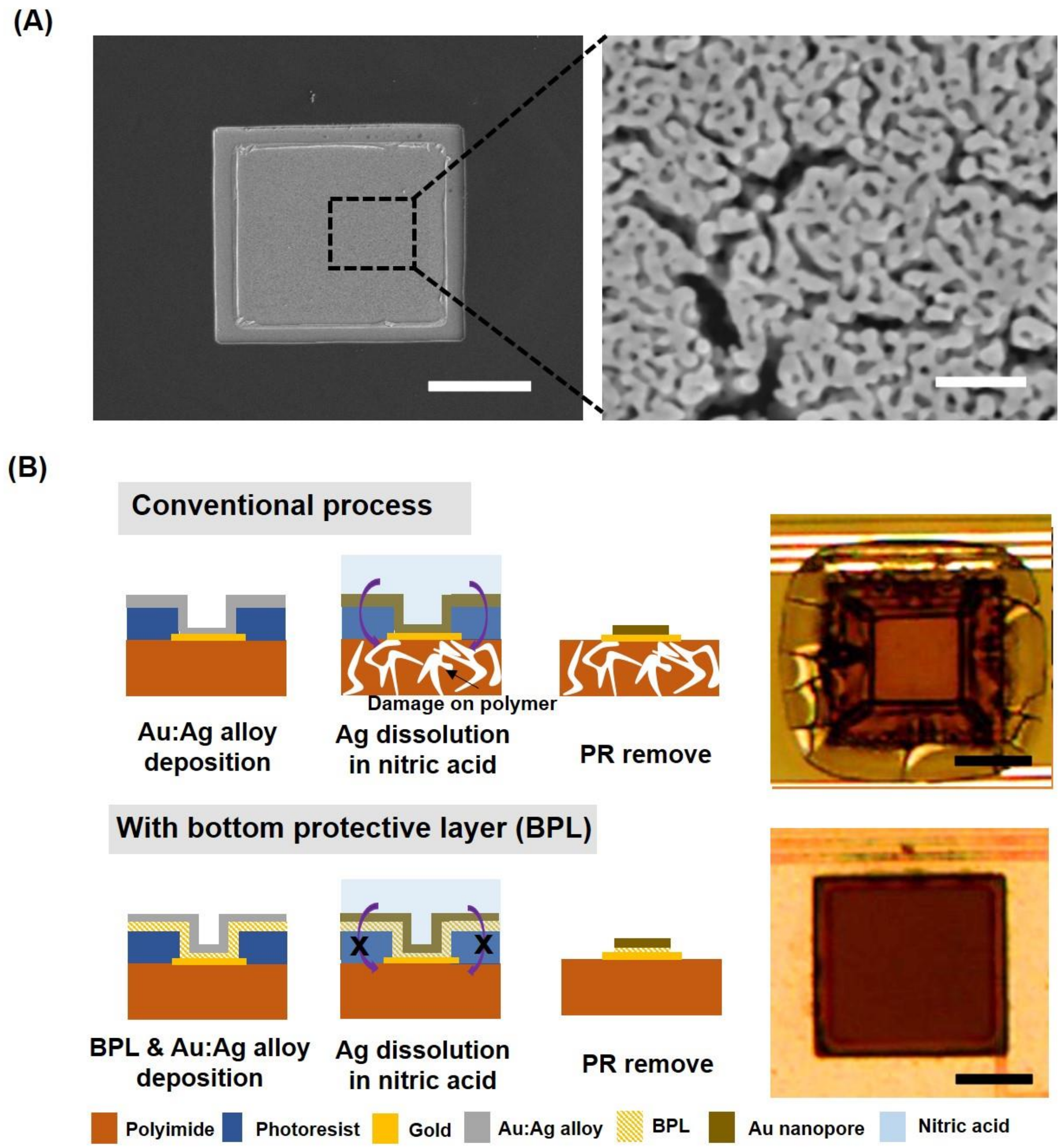

Figure S3. Characterization of gold nanoporous microelectrodes. (A) SEM image of 100 × 100 μm^2 gold nanoporous microelectrodes and magnified image. Scale bar, 50 μm (left) or 50 nm (right). (B) Comparison of the conventional nitric acid dissolution process (top), which degrades the polyimide layer, versus a process that includes a bottom protective layer (BPL) to prevent damage to the polymer (bottom). Righthand panels show corresponding microscopic images of the resulting microelectrodes. Scale bar, 50 μm.

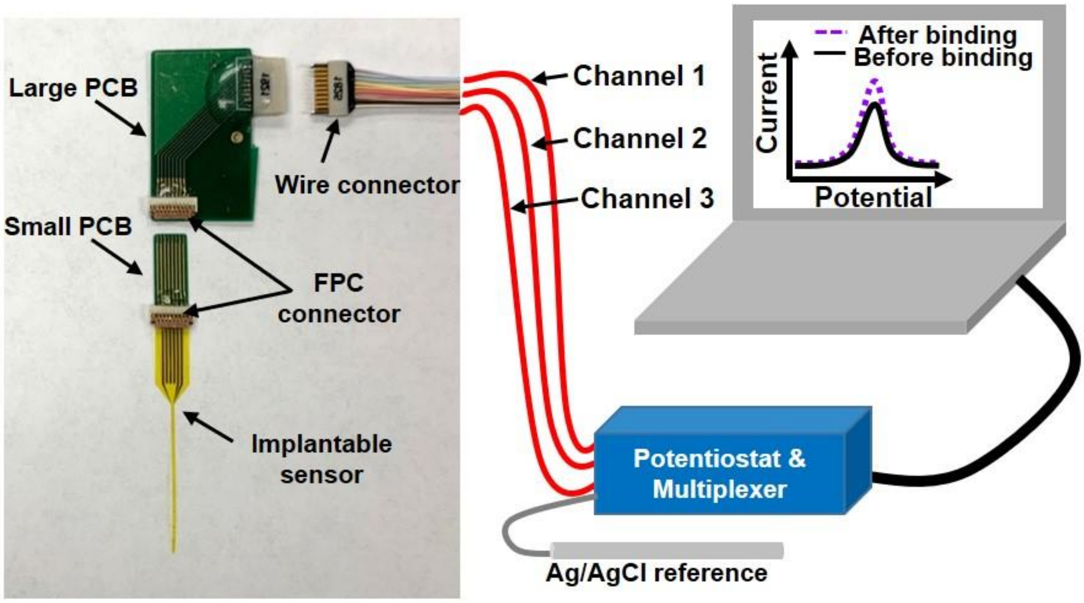

Figure S4. The electrochemical measurement system used in this study. The whole system includes the implantable sensor, FPC connector and printed circuit board (PCB) connection, and commercial potentiostat together with Ag/AgCl reference electrode, connected to a computer with custom Matlab code for real-time data processing and visualization. Photo Credit: Ji-Won Seo, Stanford University.

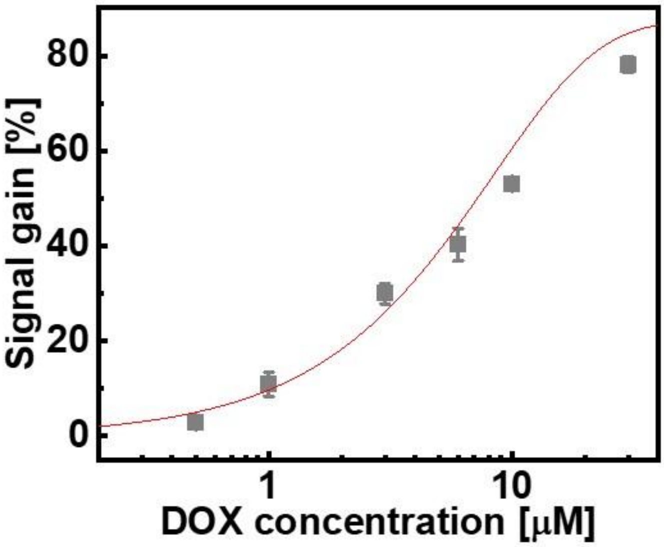

Figure S5. Calibration curve fitted to the average signal gain calculated from the multi-channel data shown in Figure 2B. Error bars were calculated from the three-channel data at each concentration.

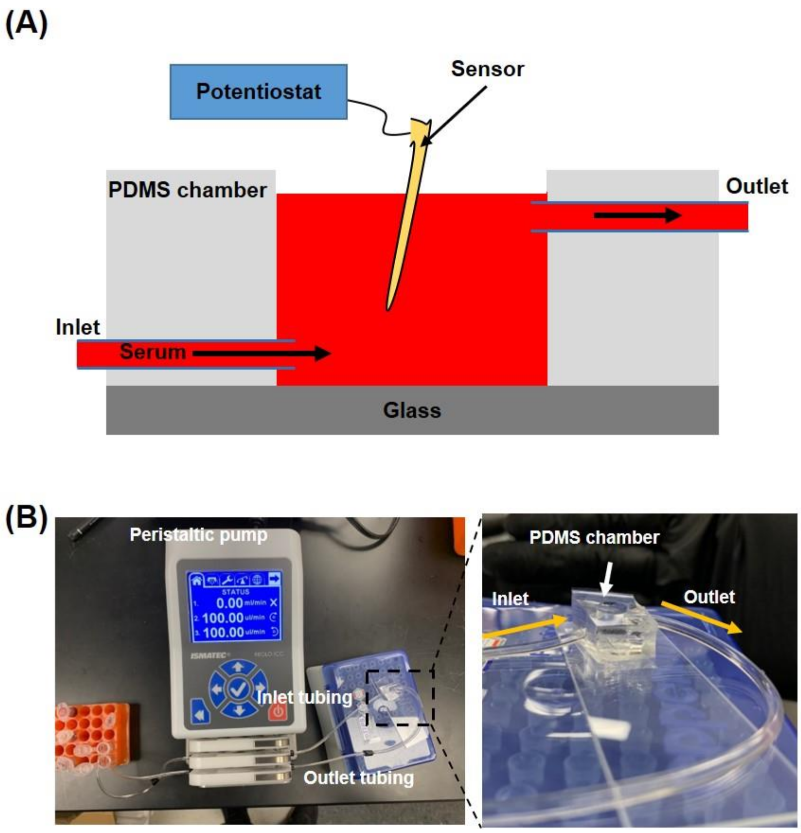

Figure S6. Scheme (A) and photos (B) of the system used for testing our sensor in flowing serum. Photo Credit: Ji-Won Seo, Stanford University.

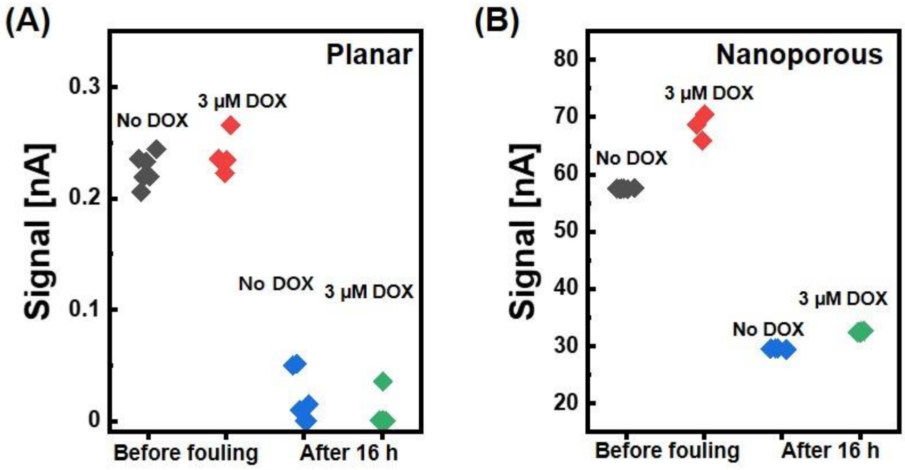

Figure S7. Raw signal before and after spiking 3 μM DOX into flowing serum for (A) planar and (B) nanoporous gold microelectrodes. Data are shown for initial signal before biofouling can occur, and for signal obtained after 16 h in blood serum.

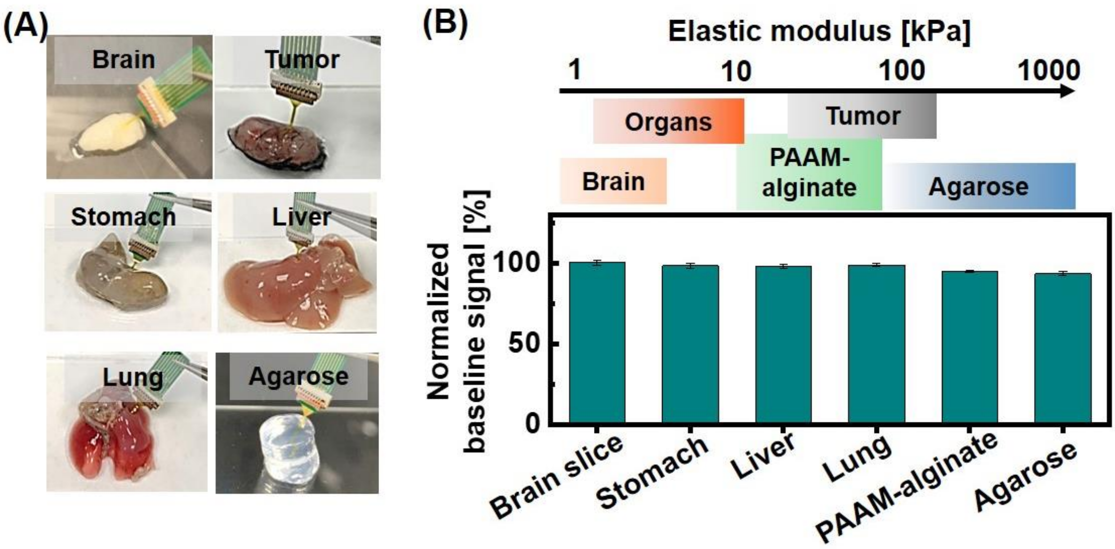

Figure S8. The mechanical characterization of the nanoporous microelectrode device. (A) Photos of our nanoporous microelectrode sensor inserted into various biological tissues or other materials and (B) the corresponding baseline signal change after 100 insertion cycles. These various materials reflect a broad range of elastic modulus values—the various biological tissues ranged from 1 to 100 kPa, and we also included PAAM-alginate hydrogel (30 kPa) and agarose hydrogel (1,000 kPa). Photo Credit: Ji-Won Seo, Stanford University.

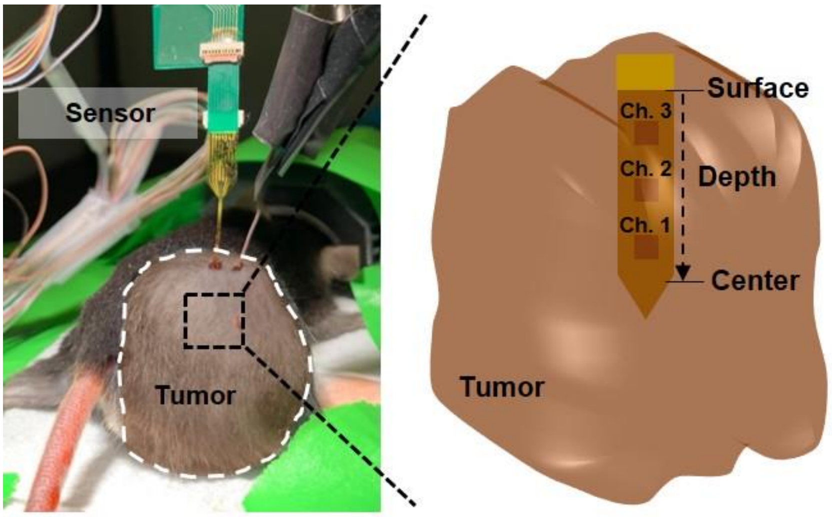

Figure S9. Microelectrode array sensor implantation into tumor tissue of an anesthetized mouse. Photo Credit: Ji-Won Seo, Stanford University.

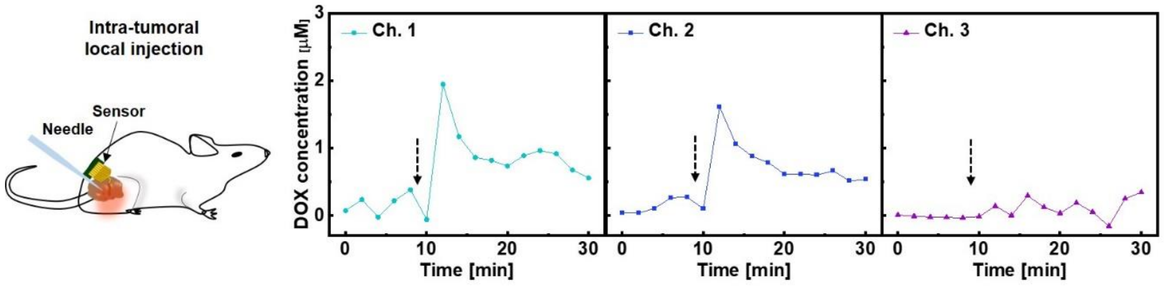

Figure S10. Real-time DOX concentration measurements at each sensor channel after intra-tumoral injection of 10 μg/g DOX near channel 1 at t = 9 min (black dotted arrow).

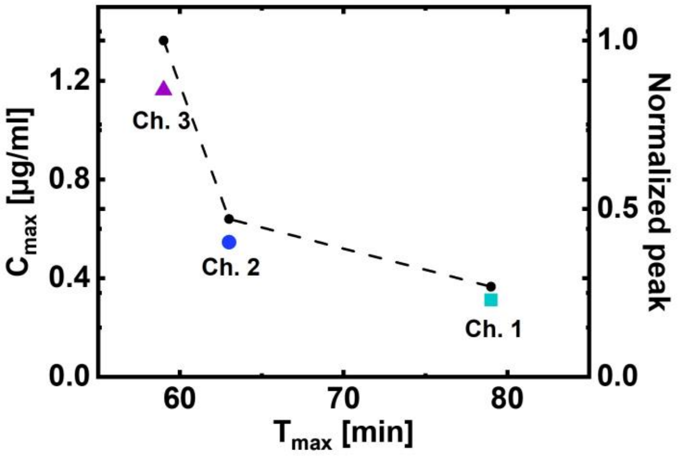

Figure S11. Maximum drug concentration (C_{max}, colored dots) and normalized peak (black dotted line) at the time at which C_{max} was reached (T_{max}) at the three sensor channels.

Table S1. Comparison of previous DOX measurements in tissue.

Works	Tumor model	Sacrificing animals	Sample preparation	Measurement type	DOX injection volume & Max DOX concentration
(57)	Orthotopic ovarian cancer	Multiple animals per each time point detection.	Tissue extraction, centrifuged, stored at 4ºC, washing	Multiple time points, *Ex vivo*	10 µg/g DOX injection, 8 µg/g DOX in tumor
(58)	Tumor-free	Multiple animals per each time point detection.	Tissue extraction, centrifuged	Multiple time points, *Ex vivo*	5 µg/g DOX injection, 4 µg/g DOX in liver tissue
(59)	Tumor-free	Multiple animals per each time point detection.	Tissue extraction, centrifuged, stored overnight	Multiple time points, *Ex vivo*	12 µg/g DOX injection, ~2 µg/g DOX in muscle
This work	Melanoma	No	Don't need	Real-time, *In vivo*	10 µg/g DOX injection, ~1.05 µg/g DOX in tumor
